# Causal relationship between childhood obesity and osteoporosis: A STROBE two-sample Mendelian randomization study

**DOI:** 10.1097/MD.0000000000041209

**Published:** 2025-01-03

**Authors:** Chaoshun Zheng, Taiqiu Chen, Longsheng Zhang, Chuchun Lin, Xuhui He

**Affiliations:** a Department of Orthropedics II, Jieyang People’s Hospital, Jieyang, China; b Department of Anesthesiology, Jieyang People’s Hospital, Jieyang, China.

**Keywords:** childhood obesity, Mendelian randomization, osteoporosis

## Abstract

The causal relationship between childhood obesity and osteoporosis is not yet clear. Two-sample randomized Mendelian analysis was applied to examine the causal relationship between childhood obesity and osteoporosis. This study employs a two-sample Mendelian randomization (MR) approach. The single-nucleotide polymorphisms associated with childhood obesity and summary-level data for osteoporosis were selected from publicly published genome-wide association study. The childhood obesity dataset includes individuals under the age of 18 with a body mass index exceeding the 95th percentile, representing both male and female European children. The osteoporosis dataset includes individuals with osteoporosis from the European population (age 0–70), encompassing both genders. MR analysis was primarily conducted via inverse-variance weighted analysis. Quality of our study was assessed according to STROBE-MR guidelines. MR analysis revealed a statistically significant association between childhood obesity and osteoporosis via the inverse-variance weighted method (odds ratio 0.9985, 95% CI [0.9974, 0.9996], *P* = .0087). Other MR analysis methods also confirmed this result. The heterogeneity analysis and sensitivity analysis show the accuracy and robustness of our results. Our MR study revealed a significant causal relationship between childhood obesity and osteoporosis, indicating that childhood obesity can reduce the incidence of osteoporosis.

## 1. Introduction

Osteoporosis is characterized by a reduction in bone mass, deterioration of the bone microstructure, and susceptibility to fragility fractures. As the population ages, osteoporosis has become a significant health issue affecting human society. More than 50% of postmenopausal White women will experience at least 1 osteoporosis fracture, whereas only 33% of patients who suffer a hip fracture can regain their ability to live independently.^[1]^ Failure to address osteoporosis in advance can lead to high mortality and morbidity. It is highly important to explore the causes of osteoporosis and intervene early. Research has identified factors contributing to osteoporosis, including low body mass index (BMI), low blood calcium levels, and insufficient outdoor activity time.^[2]^

The relationship between BMI and osteoporosis has been the subject of numerous studies. John P Kemp and colleagues conducted a Mendelian analysis to examine the link between obesity and bone mineral density (BMD) at various body sites in children. They reported that obesity can lead to increased bone density in various parts of the body, such as the head, upper limbs, lower limbs, spine, and pelvis, with this effect being more pronounced in weight-bearing areas.^[3]^ Julian V and colleagues found that in adolescents, male hip bone mineral density is positively correlated with BMI-percentile, and in both male and female populations, lumbar spine bone mineral density is positively correlated with body weight.^[4]^ Evensen E and colleagues’ cohort study found that obesity at age 16.5 years is associated with higher total hip and total body BMD.^[5]^ Given the large number of individuals with a history of childhood obesity, studying their incidence of osteoporosis could be beneficial for the formulation of prevention and treatment strategies for this population.

Childhood obesity has emerged as a significant public health concern in developed countries. It can impact children’s growth and development, limit their physical ability, harm their psychological well-being, increase the risk of chronic diseases such as stroke and diabetes, and impose a substantial social burden. Approximately 1 in 5 American children are diagnosed with obesity.^[6]^ There is a considerable body of research on the skeletal development of obese children, yet the findings are often contradictory. Some studies indicate that childhood obesity increases the risk of limb fractures, illustrating the adverse effects of childhood obesity on bone density,^[7]^ and this impact can also lead to a decrease in bone density after puberty.^[8]^ However, some studies show that obese children have increased bone density and bone strength,^[9,10]^ including the 2 studies mentioned in the text above, which also demonstrate this positive effect.^[4,5]^ This inconsistency may stem from the influence of various confounding factors, which undermines the reliability of the conclusions.

This study employs Mendelian randomization (MR) analysis to investigate the causal relationship between childhood obesity and the occurrence of osteoporosis. As a conventional statistical technique, MR effectively assesses the association between exposure and outcome, mitigating the impact of confounding variables and the problem of reverse causality.^[11]^ This method is especially valuable in fields with numerous confounding factors. In research fields where randomized clinical trial (RCT) studies are lacking, Mendelian studies achieve research subjects by selecting single-nucleotide polymorphisms (SNPs), which, similar to the random assignment in RCTs, can achieve effects comparable to those of RCT research.

In summary, this study aims to determine the causal relationship between childhood obesity and osteoporosis through Mendelian analysis, providing a foundation for the diagnosis, treatment, and prevention strategies for the relevant population.

## 2. Materials and methods

### 2.1. Study design

This research is conducted via a two-sample MR analytical approach. By utilizing large-sample genome-wide association study (GWAS) data that are publicly accessible, this method identifies the exposure and outcome factors, thus examining their causal relationships while effectively mitigating biases. The study is predicated on 3 foundational assumptions: (1) the relevance assumption is that the instrumental variables (IVs) are directly correlated with the exposure factor. (2) The assumption of independence posits that the selected IVs are unrelated to any confounding variables that may influence the relationship between the exposure and the outcome. (3) The assumption of exclusion restriction asserts that the IVs have no direct link to the outcome, with their influence being mediated solely through the exposure factor.^[12]^

**Figure 1.**
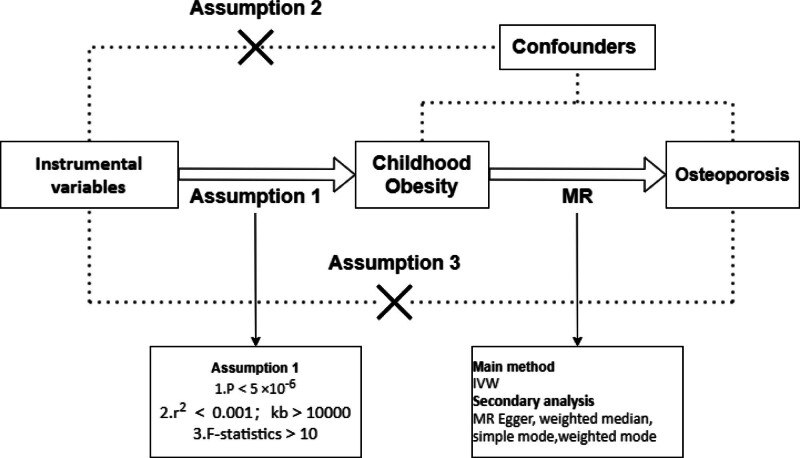
Flow diagram of this Mendelian randomization (MR) study. IVW = inverse-variance weighted. (1) The relevance assumption is that the instrumental variables (IVs) are directly correlated with the exposure factor. (2) The assumption of independence posits that the selected IVs are unrelated to any confounding variables that may influence the relationship between the exposure and the outcome. (3) The assumption of exclusion restriction asserts that the instrumental variables have no direct link to the outcome, with their influence being mediated solely through the exposure factor.

The flowchart of this study is summarized in Figure 1. In brief, childhood obesity is considered an exposure factor, whereas osteoporosis is considered an outcome factor. By applying rigorous inclusion and exclusion criteria, SNPs associated with childhood obesity are selected as IVs. The causal relationship between childhood obesity and osteoporosis was explored via a two-sample MR approach. A series of sensitivity analyses are conducted to validate the robustness of the relationship. A quality evaluation was performed, focusing on compliance with the guidelines set by the Strengthening the Reporting of Mendelian Randomization Studies (STROBE-MR).

### 2.2. Data source

In this work, to address potential biases stemming from ethnic diversity, we exclusively utilized GWAS data from European populations. The data pertaining to childhood obesity are sourced from a GWAS conducted by the Early Growth Genetics consortium (GWAS ID: ieu-a-1096).^[13]^ This study encompasses 5530 cases (BMI exceeding the 95th percentile) and 8318 controls (BMI below the 50th percentile). The dataset includes individuals under the age of 18 encompassing both male and female European children. A total of 2,442,739 SNPs within this dataset were identified.

The outcome measure for osteoporosis in this study is derived from a GWAS led by Handan Melike Dönertaş and colleagues, utilizing data from the UK Biobank (GWAS ID: ebi-a-GCST90038656).^[14]^ The dataset comprises 7751 cases of osteoporosis (femoral neck BMD T-score of ≤ -2.5) and 476,847 control subjects. The dataset includes individuals from European populations, ranging from 0 to 70 years old, encompassing both males and females. The comprehensive analysis identified 9,587,836 SNPs within this dataset. This study utilizes publicly available GWAS summary statistics as the data source. No new data were added to the research process; hence, no additional ethical approval was needed.

The details of the data are listed in Table 1.

**Table 1:** Data sources.

Phenotype	Sample size	nCases/nControls	Number of SNPS	Sex	Population	PubMed ID
Childhood obesity	13,848	5530/8318	2,442,739	Males and females	European	22484627
Osteoporosis	484,598	7751/476,847	9,587,836	Males and females	European	33959723

SNPs = single-nucleotide polymorphisms.

### 2.3. SNPs in exposure and outcome selection

From the aforementioned GWAS database, we selected eligible IVs. The following steps were taken to ensure that the IVs met 3 assumptions. In terms of relevance, we initially set the *P* value to be less than 5 × 10^-8^, which resulted in only 6 SNPs being selected. This did not meet the MR requirement of having at least 10 IVs,^[15]^ so we adjusted the *P* value threshold to 5 × 10^-6^, yielding a total of 15 viable SNPs. For the independence aspect, all SNPs were clumped to avoid linkage disequilibrium, with the clump window set to *r*^2^ = 0.001 and a distance of 10,000 kb. We subsequently harmonized the data, and 1 SNP (rs1040070) was removed because it is palindromic with intermediate allele frequencies. With respect to the exclusivity assumption, there was no correlation between all the SNPs and the outcome factors (*P* > 5 × 10^-6^). Finally, we calculated the statistical strength via F statistics to avoid weak instrument bias and considered F values >10 to indicate the absence of this bias.^[16]^ We verified through SNiPA (https://snipa.org/) that the selected SNPs are not related to the confounding factors associated with the outcome. Detailed information on the IVs can be found in Table 2.

**Table 2:** Detailed information on the identified SNPs in terms of exposure and outcomes.

SNP			Exposure (childhood obesity)						Outcome (osteoporosis)				F
RS ID	Chromosome	Position		EA	OA	Beta	SE	*P* value	Beta	SE	*P* value	EAF	
rs10913469	1	177913519		C	T	0.1773	0.033	7.99E-08	0.0003	0.0003	.3000	0.2059	28.8620
rs13130484	4	45175691		T	C	0.1434	0.0272	1.30E-07	-0.0006	0.0003	.0290	0.4283	48.5155
rs17697518	18	38765659		T	C	0.1855	0.0389	1.85E-06	-0.0005	0.0004	.2000	0.1246	41.8479
rs256335	19	34315896		T	C	0.1214	0.0262	3.72E-06	-0.0001	0.0003	.6500	0.4615	27.7906
rs28636	5	66149113		T	C	-0.1474	0.0316	3.07E-06	-0.0004	0.0003	.1900	0.2215	21.4006
rs4833407	4	113311790		A	C	0.1226	0.0265	3.88E-06	0.0004	0.0003	.1100	0.4119	23.2489
rs4854344	2	638144		T	G	0.2445	0.0351	3.22E-12	-0.0008	0.0003	.0210	0.8282	21.7549
rs4864201	4	130731284		C	T	-0.1355	0.0281	1.41E-06	0.0004	0.0003	.1500	0.6369	38.0602
rs571312	18	57839769		A	C	0.1986	0.0309	1.25E-10	-0.0004	0.0003	.1500	0.2377	23.3537
rs6752378	2	25150116		A	C	0.1695	0.0262	1.05E-10	0.0000	0.0003	.9600	0.4926	54.8741
rs7138803	12	50247468		A	G	0.1672	0.0271	6.50E-10	-0.0005	0.0003	.0780	0.3649	22.6773
rs9299	17	46669430		T	C	0.1343	0.0282	1.91E-06	-0.0004	0.0003	.1500	0.6459	41.3027
rs9568856	13	54064981		A	G	0.1909	0.0395	1.36E-06	-0.0003	0.0004	.4700	0.1355	22.7366
rs9941349	16	53825488		T	C	0.1978	0.0267	1.16E-13	-0.0005	0.0003	.0350	0.4063	21.4670

EA = effect allele, OA = other allele.

### 2.4. Statistical analysis

To explore the causal relationship between childhood obesity and osteoporosis, this two-sample MR analysis was conducted via R software (version 4.4.1, The R Foundation for Statistical Computing) and the two-sample MR package (version 0.6.6), along with the MR-PRESSO package for analysis. All steps of this study were conducted in accordance with STROBE statement.^[17]^ Specifically, we employed 5 distinct MR methodologies. The primary method utilized was the inverse-variance weighted (IVW) approach, which combines information from multiple genetic variants and employs a weighted calculation to increase the precision and reliability of the estimates.^[18]^ In addition to the IVW method, we also employed MR-Egger, weighted median, simple mode, and weighted mode methods for validation. A difference was considered statistically significant when the *P* value was <.05, indicating that childhood obesity could influence the outcome of osteoporosis. This causal relationship was quantified via the odds ratio (OR) and 95% confidence intervals. Heterogeneity was assessed via the Cochran Q statistic,^[19]^ and the absence of heterogeneity was assumed when the *P* value was >.05. To exclude the possibility that IVs affect the outcome through pathways other than exposure, we utilized the MR-Egger intercept^[20]^ and MR-PRESSO^[21]^ methods. The absence of pleiotropy was assumed when the *P* value was >.05. Sensitivity analysis was conducted via the “leave-one-out” method,^[22]^ ensuring that the causal relationship between the exposure and the outcome was not driven by a single SNP.

## 3. Results

### 3.1. Causal relationship between childhood obesity and osteoporosis

**Figure 2.**
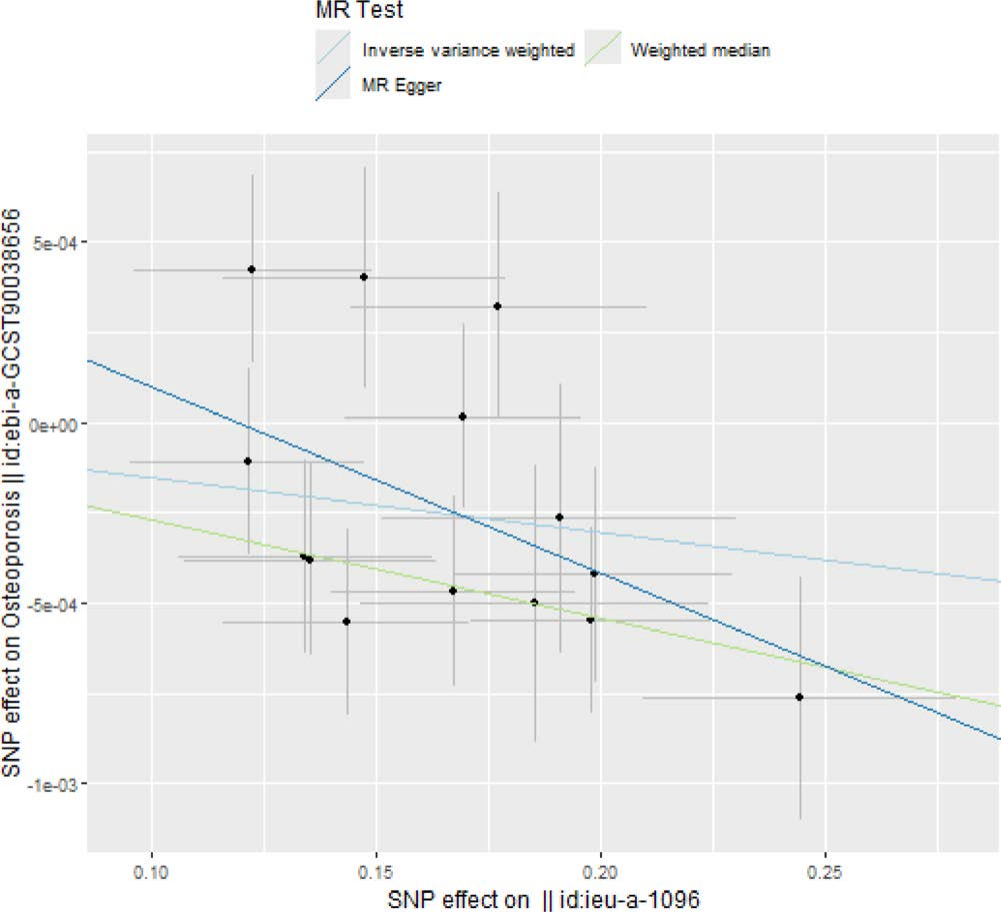
Scatterplot of childhood obesity and osteoporosis.

The IVW study revealed a statistically significant association between childhood obesity and osteoporosis (OR 0.9985, 95% CI [0.9974, 0.9996], *P* = .0087). Additionally, 4 other methods were employed to corroborate this relationship. The weighted median (OR 0.9973, 95% CI [0.9960, 0.9986], *P* < .001), simple mode (OR 0.9972, 95% CI [0.9952, 0.9993], *P* = .019), and weighted mode (OR 0.9972, 95% CI [0.9955, 0.9990], *P* = .009) all indicated statistically significant relationship between the exposure and outcome factors. The MR-Egger method (OR 0.9949, 95% CI [0.9893, 1.0004], *P* = .0949), however, yielded a *P* value of .0949 (>.05). All MR methods presented a consistent direction. In summary, the MR approach demonstrated a statistically significant relationship between childhood obesity and osteoporosis. A scatterplot of childhood obesity and osteoporosis is depicted in Figure 2.

### 3.2. Heterogeneity and sensitivity analysis

**Figure 3.**
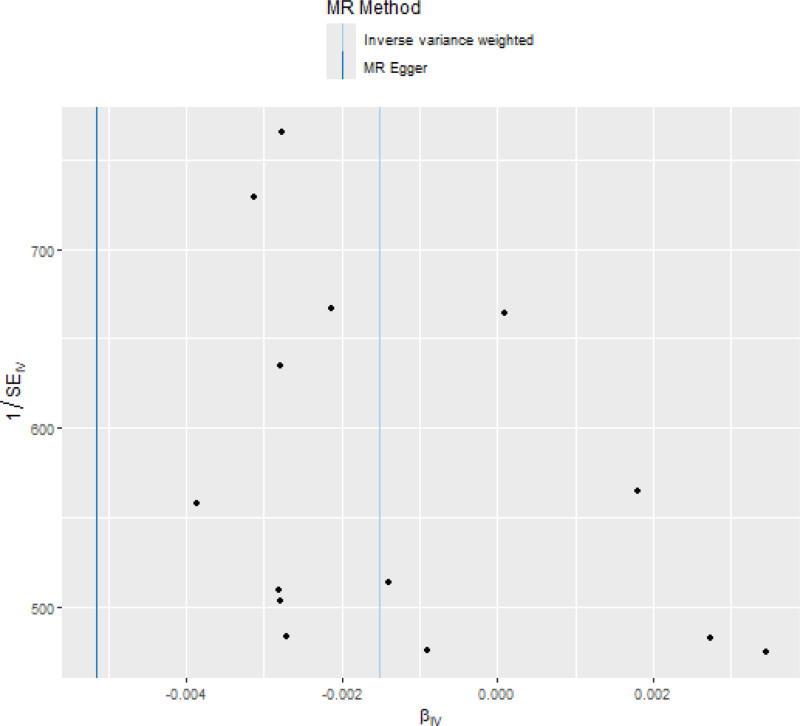
Funnel plot of childhood obesity and osteoporosis.

**Figure 4.**
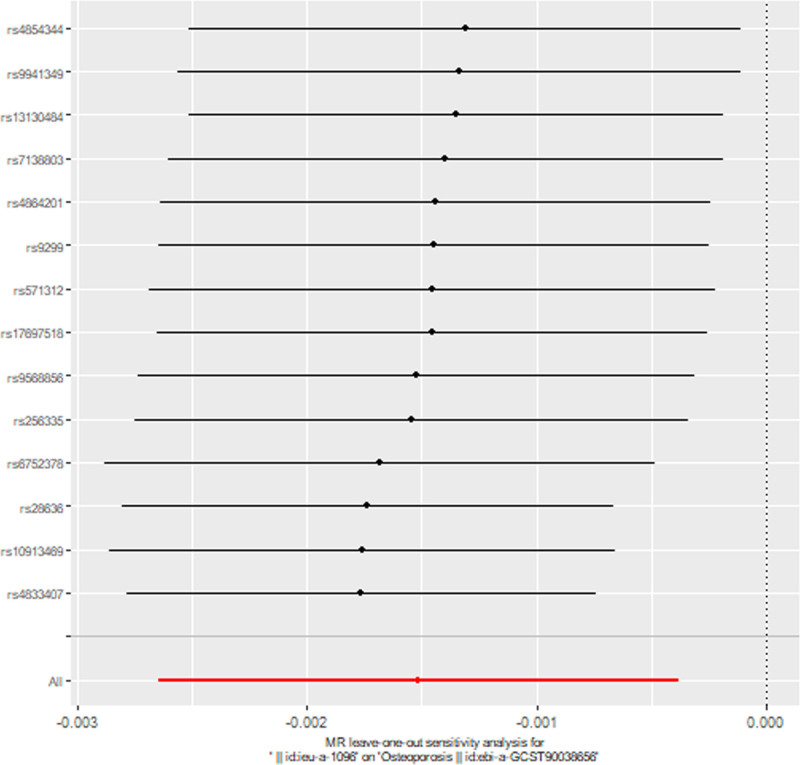
Analysis of childhood obesity and osteoporosis via the leave-one-out method.

In terms of heterogeneity, this study utilized the Cochran *Q* test, which indicated no significant heterogeneity (*P* = .0826). The heterogeneity was graphically presented via a funnel plot, as depicted in Figure 3. The absence of horizontal pleiotropy was confirmed by the MR-Egger intercept value (*P* = .2160) and MR-PRESSO analyses (*P* = .1031), which also indicated the absence of outliers. Furthermore, sensitivity analysis was conducted via a leave-one-out sensitivity test, as shown in Figure 4. No single SNP could influence the relationship, suggesting the stability and reliability of this association.

## 4. Discussion

In this study, we employed a two-sample MR approach to explore the correlation between childhood obesity and osteoporosis, and the results strongly indicated a causal relationship, suggesting that childhood obesity may reduce the incidence of osteoporosis. To our knowledge, this is the first MR analysis to investigate the relationship between childhood obesity and osteoporosis. This study provides a significant advancement in the understanding of the relationship between body weight and osteoporosis, laying a foundation for the diagnosis and treatment of osteoporosis.

Childhood obesity has become a global issue, with 18% of children and adolescents aged 2 to 19 being overweight or obese in the United States from 1999 to 2016.^[6]^ It is a public health problem affecting the majority of developed countries. This condition can lead to hypertension, cardiovascular and cerebrovascular diseases, diabetes, and other health problems.^[23]^ A previous study revealed that the incidence of obesity in adulthood among obese children is 5 times greater than that among children with a normal weight.^[24]^

The relationship between childhood obesity and bone metabolism is relatively complex. For example, research has shown that obese children are at increased risk of limb fractures, suggesting a negative impact of obesity on bone density.^[7]^ However, the increased fracture risk in obese children may also be related to its effects on mobility or gait.^[9]^ Some studies indicate that obese children have increased bone density and bone strength, suggesting a positive effect of childhood obesity on bone metabolism.^[9,10]^ This positive effect may be due to hormonal changes, but it could have a negative impact in adulthood, as evidenced by studies showing that obese children have lower bone mineral density after puberty.^[8]^

The National Osteoporosis Foundation suggests that osteoporosis in adulthood is closely related to peak bone mass during childhood, with calcium intake and physical activity being considered as factors that can promote healthy bone development.^[25]^ Peak bone mass is typically formed during the second decade of life, with about 40% of adult bone mass being shaped during the peripubertal years and maintained relatively stable throughout life.^[26]^ Cj H et al^[27]^ predicted through modeling that a 10% increase in Peak bone mass can delay the onset of osteoporosis by 13 years, which is far more significant than menopausal age, bone loss rate, etc, and concluded that a lower peak bone mass is the only important factor affecting osteoporosis in adulthood. Bone mass accrual during childhood is related to many factors, among which unchangeable factors include age, race, gender, etc, and changeable factors include physical activity, diet, obesity, etc.^[28]^

Nutrition during childhood can affect Bone mass accrual, which in turn affects peak bone mass. Adequate intake of calcium and vitamin D in the diet is an important condition for the healthy development of bones.^[25]^ Dairy products are rich in calcium and vitamin D. In a meta-analysis that included 21 RCTs, dairy consumption significantly affected bone mass in different parts of the body.^[29]^ Long-term lack of dairy intake can lead to smaller stature and lower bone density in developing children,^[30]^ and even increase the probability of fractures.^[31]^ In studies related to dietary patterns,^[32]^ children, adolescents, and young adults who follow the American dietary guidelines have higher bone density. In a study of Polish children, girls aged 3 to 7 who followed the Mediterranean diet pattern had better bone properties.^[33]^ A study using the National Health and Nutrition Examination Survey database, which included 2994 children and adolescents aged 8 to 19, found that people who consumed multiple antioxidants in their diet had higher bone density.^[34]^ The intake of more ultra-processed foods, as a current dietary trend and cause of obesity,^[35]^ has been proven in a study of a rural Ecuadorian community to possibly cause stunted growth of bone.^[36]^

As mentioned in the previous text, obese children have an increased probability of fractures,^[7]^ but their bone density and bone strength increase^[9,10]^; the relationship between the 2 is yet to be explored. The mechanisms by which childhood obesity affects bone density are currently hypothesized as follows^[37]^: (1) both adipocytes and osteoblasts originate from mesenchymal stem cells. Obesity can lead to the differentiation of mesenchymal stem cells into adipocytes and reduce the number of osteoblasts. (2) In an obese state, reduced physical activity may increase the levels of inflammatory cytokines such as TNF-alpha and interleukins, thus activating the RANKL pathway and leading to increased bone resorption. (3) The diet of obese children is rich in fat, which can lead to impaired calcium resorption. (4) Obesity status increases bone loading, which mechanically stimulates bone growth.

However, current research is largely based on clinical studies, which do not effectively eliminate the influence of confounding factors, resulting in contradictory conclusions. Cellular and animal experiments also cannot holistically explore the relationship between childhood obesity and bone health. This study, through MR studies and the screening of IVs, explores the causal relationship between childhood obesity and osteoporosis in adulthood from a genetic perspective, excluding the influence of confounding factors, thereby providing a solid foundation for further research.

The prevalence of osteoporosis is lower among people with a history of childhood obesity, allowing for a reduced frequency of screening for this group. Instead, the focus can be shifted toward other critical health concerns, including hypertension, cardiovascular diseases, cerebrovascular diseases, and diabetes, to make efficient use of the limited medical resources. Nonetheless, the presence of additional risk factors still necessitates prompt management of osteoporosis. The potential mechanisms linking childhood obesity with osteoporosis were not investigated in this study, and future studies could explore the relevant mediating mechanism.

This study employed two-sample MR analysis, with both exposure and outcome factors derived from GWAS, ensuring a large sample size and robust statistical power. MR allows for the analysis of causal relationships and effectively excludes the influence of confounding factors, achieving an effect that traditional observational studies cannot match. Through heterogeneity and sensitivity analyses, we confirmed the reliability and robustness of our analytical results. However, this study has several limitations. First, the selection criteria for IVs were adjusted from an initial threshold of 5 × 10^-8^ to 5 × 10^-6^ due to insufficient variables, which may increase the likelihood of false positives and weak instrumental variable bias. However, we calculated F statistics to assess the risk of such bias and found that all SNPs had F values >10. Second, the study population is limited to individuals of European descent, restricting our result to extrapolate to other ethnic groups.

## 5. Conclusion

Our MR study revealed a significant causal relationship between childhood obesity and osteoporosis, indicating that childhood obesity can reduce the incidence of osteoporosis. However, further studies are needed to examine the biological mechanisms underlying this association.

## Acknowledgments

We appreciate all investigators for providing publicly summary data.

## Author contributions

**Conceptualization:** Chaoshun Zheng.

**Data curation:** Chaoshun Zheng, Taiqiu Chen, Longsheng Zhang, Chuchun Lin.

**Formal analysis:** Chaoshun Zheng, Taiqiu Chen, Longsheng Zhang.

**Investigation:** Chaoshun Zheng, Taiqiu Chen, Longsheng Zhang.

**Methodology:** Chaoshun Zheng, Taiqiu Chen, Longsheng Zhang.

**Resources:** Xuhui He.

**Software:** Chaoshun Zheng, Longsheng Zhang, Xuhui He.

**Visualization:** Chaoshun Zheng.

**Writing – original draft:** Chaoshun Zheng, Taiqiu Chen, Longsheng Zhang, Chuchun Lin.

**Writing – review & editing:** Xuhui He.
